# Retreatment and Outcomes of Recurrent Intracranial Vertebral Artery Dissecting Aneurysms after Stent Assisted Coiling: A Single Center Experience

**DOI:** 10.1371/journal.pone.0113027

**Published:** 2014-11-13

**Authors:** Ying Song, Yang Wang, Chuanhui Li, Yanmin Wang, Shiqing Mu, Xinjian Yang

**Affiliations:** Department of Interventional Neuroradiology, Beijing Neurosurgical Institute and Beijing Tiantan Hospital, Capital Medical University, Beijing, China; St Michael's Hospital, University of Toronto, Canada

## Abstract

**Background and purpose:**

The retreatment of recurrent intracranial vertebral artery dissecting aneurysms (VADAs) after stent assisted coiling (SAC) has not yet been studied. The purpose of this study was to evaluate the strategies and outcomes for retreatment of recurrent VADAs after SAC.

**Methods:**

Between September 2009 and November 2013, six consecutive patients presenting with recurrent intracranial VADAs after SAC were enrolled in this study. They were all male with age ranging from 29 to 54 years (mean age, 46.2 years). The procedures of treatments and angiographic and clinical follow-up were reviewed retrospectively. Retreatment modalities were selected individually according to the characteristics of recurrence. The outcomes of retreatment were evaluated by angiographic and clinical follow-up.

**Results:**

Six patients with recurrent intracranial VADAs after SAC were retreated, with second SAC in three patients, coil embolization, double overlapping stents placement and endovascular occlusion with aneurysm trapping in one patient, respectively. Immediate angiographic outcomes of retreatment were: complete occlusion in three patients, nearly complete occlusion in two patients, and contrast medium retention in dissecting aneurysm in one patient. All cases were technically successful. No complications related to endovascular procedures occurred. Angiographic follow-up was available in all five patients treated with second SAC or double overlapping stents, which was complete occlusion in four patients, obliteration of parent artery in one patient, showing no recurrence at 4–11 months (mean: 8.6 months). Clinical follow-up was performed in all six patients at 11–51 months after initial endovascular treatment and at 9–43 months after retreatment. The mRS of last clinical follow-up was excellent in five patients and mild disability in only one patient.

**Conclusions:**

Endovascular retreatment is feasible and effective for recurrent intracranial VADAs after SAC. Individualized strategies of retreatment should be enacted according to the characteristics and reasons for the recurrence.

## Introduction

Intracranial vertebral artery dissecting aneurysms (VADAs) have been increasingly recognized as a cause of subarachnoid hemorrhage (SAH) and posterior circulation ischemia with high morbidity and mortality, especially in young and middle-aged people [1]–[4]. In recent years, endovascular treatment has become one of the major modalities of treatment for these lesions, including deconstructive (proximal occlusion, endovascular occlusion with or without aneurysm trapping) and reconstructive treatment (stent assisted coiling, SAC, stent only) [5]–[6]. Endovascular occlusion with or without aneurysm trapping of dissected segment of the parent artery is deemed as the most reliable technique to prevent re-bleeding, but not applicable for VADAs involving the posterior inferior cerebellar artery (PICA) origin, bilateral vertebral artery (VA), predominant VA and the basilar artery [7]. SAC has become a vital option for the endovascular treatment for VADAs, which not only allows preserving the parent artery, but also occludes the dissecting aneurysm [8]–[13]. With the increasing number of cases being treated, recurrence after SAC is gradually seen, [4], [14]–[17] and some patients with recurrent intracranial VADAs need retreatment. However, the retreatment and outcomes of recurrent intracranial VADAs after SAC have rarely been studied [19], [20].

The present study is to retrospectively analyze the reasons of recurrence, evaluate the strategies and outcomes of retreatment for recurrent intracranial VADAs after SAC in order to improve the efficacy of endovascular treatment for intracranial VADAs. We especially pay attention to the outcomes of retreatment. We wonder if recurrence will happen again after retreatment as seen post initial endovascular treatment. To our best knowledge, it is the first report about the retreatment and follow-up outcomes of recurrent VADAs after SAC.

## Methods

### Patients

The retrospective study was approved by Ethics Committee at Beijng Tiantan Hospital affiliated to the Capital Medical University of China. Written Informed consents were obtained from patients or their family members.

Between September 2009 and November 2013, 112 consecutive patients presenting with intracranial veterbrobasilar dissecting aneurysms (VBDAs) underwent endovascular treatment including endovascular occlusion with or without aneurysm trapping, SAC and stent only, at our institution. 72 patients were treated with SAC. Angiographic follow-ups were available in 59 patients. Recurrence was confirmed by angiographic follow-up in 10 patients. Retreatment needed to be performed in six patients with VADAs, which is the focus of our study.

All 6 patients were male, with age ranging from 29 to 54 years (mean age, 46.2 years). The lesions were located in the left VA (cases 2, 5), right VA (cases 1, 3, 4, 6), and involving PICA (cases 1, 3, 4). MR showed intramural hematoma (cases 1–6), intimal flap (cases 4, 5) and mass effect (cases 3, 6). Angiographic signs included fusiform dilation (cases 1, 2, 4, 6); pearl and string sign (case 3) and double lumen sign (case 5). The clinical status on admission was evaluated by the modified Rankin Scale (mRS). Demographic, angiographic and clinical characteristics of these six patients are shown in Table 1.

**Table 1:** Demographic, angiographic and clinical characteristics on admission.

Patient NO.	Age(yrs)/sex	Initial symptom	MRI findings	DSA findings		mRS
				Angiographic sign	lesion site	
1	43/M	IVH, SAH	IMH	fusiform dilation	right VA involving PICA	2
2	49/M	headache	IMH	fusiform dilation	left VA distal to PICA	2
3	29/M	dizziness, hemiplegia	ME, IMH	pearl and string sign	right VA involving PICA	1
4	49/M	headache, dizziness	IMH,IF	fusiform dilation	right VA involving PICA	1
5	53/M	headache, dizziness	IMH, IF	double lumen sign	left VA proximal to PICA	2
6	54/M	headache, dysphagia	ME, IMH	fusiform dilation	right VA distal to PICA	3

IVH = intraventricular hemorrhage; SAH = subarachnoid hemorrhage; VA = vertebral artery; PICA = posterior inferior cerebellar artery; IMH = intramural hematoma; IF = Intimal flap; ME = Mass effect.

### Initial SAC and follow-up

All procedures were performed with patients under general anesthesia. After femoral sheath placement, systemic heparinization was started with a loading dose of 3000 IU of heparin and was maintained with a dose of 1000 IU on an hourly basis. Through the guiding catheters, proper shaped microcatheters were introduced over microguidewires and navigated into the dissecting aneurysms. Stents were deployed after the microcatheter used for coiling was in position and the first coil was deployed but not detached (jailing technique). Bare coils were used including Guglielmi detachable coils (Stryker Neurovascular, Fremont, California, USA), NXT coils (Covidien/ev3, Irvine, California, USA), Complex and HyperSoft coils (Microvention Terumo, Tustin, California, USA). Enterprise stent (Cordis Codman and Shurtleff Inc, Raynham, Massachusetts, USA) was used in all six patients. Five patients (cases 1–5) were treated using a single stent assisted coiling procedure, and one patient (case 6) was treated with three overlapping stents and coils. Patients received 100 mg of aspirin orally and 75 mg of clopidogrel daily for three days preoperatively and for one month postoperatively, then aspirin monotherapy was continued for 6 months after the endovascular procedure.

The extent of occlusion of dissecting aneurysms was defined as follows: complete occlusion where the entire dissecting aneurysm sac was occluded; nearly complete occlusion where most of the dissecting aneurysm sac was occluded but a small portion of the sac opacified with contrast medium; and partial occlusion where the dissecting aneurysm sac was opacified [7].

Angiographic recurrence was defined as a substantial increase in the contrast medium–filled portion of the dissecting aneurysm compared with a control angiogram taken immediately after the initial treatment [4]. Recurrences were divided into recanalization and regrowth. Recanalization means opening of the previously embolized dissecting aneurysms, which has the same size, but the coils have been moved away from the original site due to blood flow and consequent compaction. Regrowth signifies that the dissecting aneurysm has become larger and the coil mass is no longer sufficient to obliterate it [20]. Extent of occlusion and criteria of recurrence were evaluated by three neuroradiologists at our institution.

The immediate angiographic outcomes of initial treatment were complete occlusion (cases 2, 6), nearly complete occlusion (cases 1, 3), and partial occlusion (cases 4, 5). Recurrence was confirmed by angiographic follow-up at 2 to 8 months (mean: 5.2 months). According to the angiographic characteristics, recurrence was manifested as recanalization in two patients (cases 3, 5), regrowth in four patients (cases 1, 2, 4, 6).

The symptoms of recurrence were headache in four patients (cases 1, 2, 4, 5), sudden dyspnea in one patient (case 6). There were no obvious symptoms before recurrence seen on angiographic follow-up in one of the patient (case 3). Initial endovascular treatment results, recurrence type, time, symptom and mRS are showed in Table 2.

**Table 2:** Initial endovascular treatment results and angiographic and clinical follow-up.

Patient NO.	Initial treatment modality	Stent type, size (mm×mm) (number)	Immediate angiographic outcomes	Angiographic FU		Clinical FU	
				Recurrence type	time (mons)	Recurrence symptom	mRS
1	SAC	EN, 4.5×28 (1)	NC	regrowth	**8**	headache	**1**
2	SAC	EN, 4.5×22 (1)	CO	regrowth	**7**	headache	**1**
3	SAC	EN, 4.5×28 (1)	NC	recanalization	**3**	no	**0**
4	SAC	EN, 4.5×37 (1)	PO	regrowth	**6**	headache	**1**
5	SAC	EN, 4.5×37 (1)	PO	recanalization	**5**	headache	**1**
6	Multi-stents assisted coiling	EN, 4.5×28 (2), 4.5×22 (1)	CO	regrowth	**2**	dyspnea	**5**

SAC = stent assisted coiling; FU = follow up; EN = Enterprise; CO = complete occlusion; NC = nearly complete; PO = partial occlusion.

### Retreatment of recurrent VADAs and follow up

The indications for retreatment must meet one of the following criteria: 1) symptoms related to SAH or posterior circulation ischemia; 2) recanalization more than 20% of the original dissecting aneurysms and 3) obvious regrowth. Retreatment modalities were selected individually according to the characteristics of recurrence which included SAC, double overlapping stents placement, coil embolization and endovascular occlusion with aneurysm trapping. Angiographic follow-up were performed between 4 and 11 months. Clinical follow-up by telephone or out-patient interview was conducted up to November 2013. The clinical status was evaluated by mRS.

## Results

**Figure 1:**
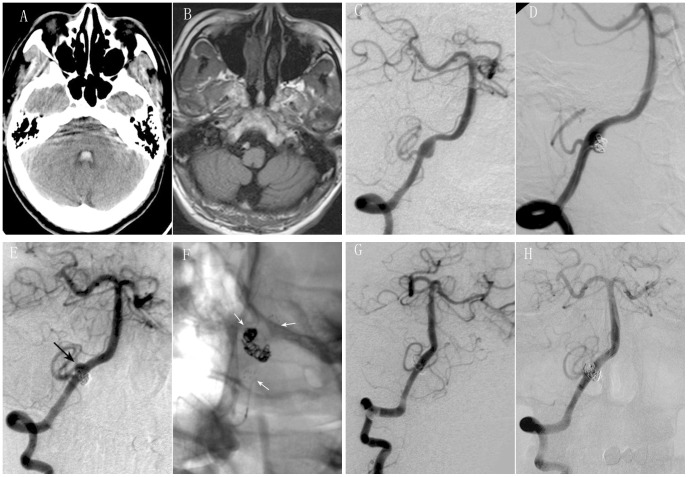
Retreatment with a second stent assisted coiling was performed for recurrent intracranial VADA after SAC. A 43-year old male presented with severe headache. CT scan showed subarachnoid hemorrhage and intraventricular hemorrhage (A). MR imaging showed intramural hematoma (B). Right vertebral angiograms of oblique view showed a dissecting aneurysm involving PICA (C). SAC were performed with near complete occlusion (D). Follow up angiography after eight months revealed regrowth of dissecting aneurysm on the opposite side and in a different location from the original dissecting aneurysm (black arrow) (E). Retreatment by SAC (white arrows) (F) was performed with complete occlusion (G). Follow up angiography after ten months of retreatment showed not only complete occlusion of dissecting aneurysm, but also patency of parent artery (H).

In three patients (cases 1, 2, 4), the recurrence was manifested as regrowth in parent arteries on the opposite side and in a different location from the original dissecting aneurysm (case 1, Fig. 1 E), from distal to the original dissecting aneurysm (case 2) or as enlargement of the original dissecting aneurysm (case 4). We believed the recurrence was due to stent malapposition with inadequate radial force against vessel wall and inability for the flow to divert. So, retreatment with a second stent assisted coiling was performed in these 3 patients (Fig. 1 F). Immediate angiographic outcomes of retreatment were: complete occlusion in one case (case 1) and nearly complete occlusion in 2 cases (cases 2, 4).

**Figure 2:**
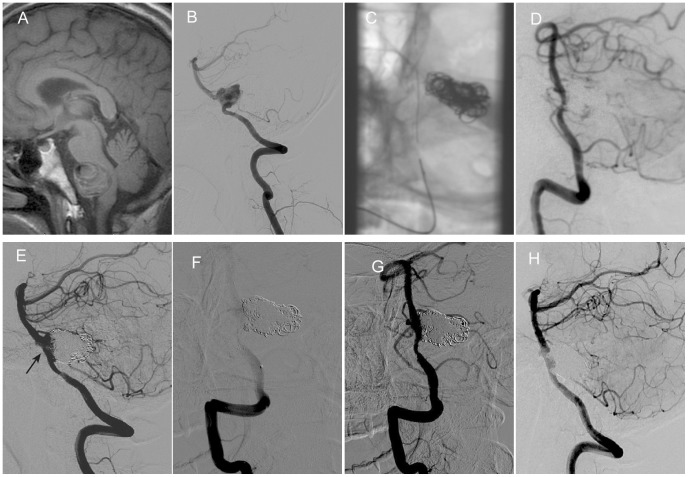
Retreatment with coil embolization was performed for recurrent intracranial VADA after SAC. A 29-year old male presented with dizziness and hemiplegia. Sagittal view of MR imaging showed intramural hematoma and compression to brain stem (A). Right vertebral angiograms showed a dissecting aneurysm involving PICA (B). SAC (C) were performed with nearly complete occlusion (D). Follow up angiography after three months revealed recanalization of the dissecting aneurysm (black arrow) (E). Retreatment was performed by coil embolization (F, G). Follow up angiography after 7 months of retreatment showed complete occlusion of right vertebral dissecting aneurysm (H).

In case 3, recurrence was shown as recanalization of previously embolized dissecting aneurysms (Fig. 2 E). The volume of dissecting aneurysm did not enlarged continuously. The reason of recurrence was due to flow disruption and consequent coil compaction, which was similar to recurrence of the sac aneurysm. So, in this case, we performed coil embolization for the recanalization. Complete occlusion was achieved as seen on immediate angiography.

In case 5, recurrent VADA was a fusiform dilation. Because of the worry of coil protrusion into the parent artery, we only deployed two Enterprise stents in an overlapping fashion trying to heal the dissection by increasing the flow diversion. Immediate angiographic outcome of the retreatment was contrast medium retention in dissecting aneurysm.

**Figure 3:**
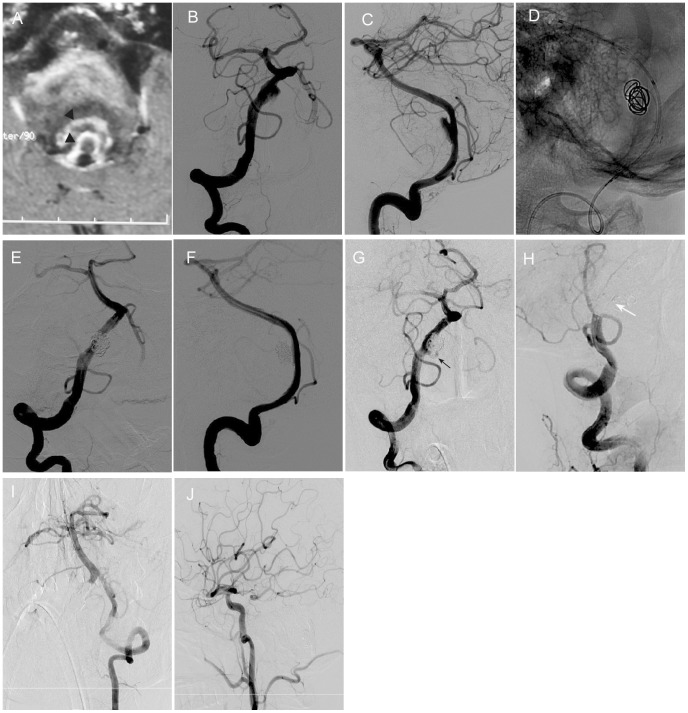
Retreatment with endovascular occlusion with aneurysm trapping was performed for recurrent huge intracranial VADA after multiple stents assisted coiling. A 54-year old male presented with headache and dysphagia. MR imaging showed huge onion skin-like thrombus (black arrowhead), intramural hematoma and compression to brain stem (A). Right vertebral angiograms of frontal (B) and lateral (C) view showed a dissecting aneurysm. Multiple stents assisted coiling (D) were performed with complete occlusion (E, F). Follow up angiography (G) after three months revealed regrowth of original dissecting aneurysm (black arrow). Retreatment by endovascular occlusion with aneurysm trapping (white arrow) (H) was performed. Collateral blood flow could be seen from contralateral VA (I) and left posterior communicating artery (J).

In case 6, a huge dissecting aneurysm accompanying an “onion skin” like thrombus causes obvious mass effect to the brain stem. Initial treatment was performed using three overlapping stents assisted coiling. Patient presented with sudden dyspnea after two months. He required emergent intubation for airway protection. His respiration was dependent of breathing machine. An immediate digital subtraction angiography revealed regrowth of coiled dissecting aneurysm (Fig. 3 G). Endovascular occlusion with aneurysm trapping was performed as the retreatment modality. Immediate contrast angiography showed complete occlusion of the parent artery (Fig. 3 H). Collateral blood flow could be seen from contralateral VA (Figure 3 I) and left posterior communicating artery (Figure 3 J).

All procedures were technically successful. No complications related to endovascular procedures.

Angiographic follow-ups were available in all five patients treated with second SAC or double overlapping stents between 4 to 11 months after retreatment. Clinical follow-ups were performed in all six patients at 11–51 months after initial endovascular treatment, and at 9–43 months after retreatment. Angiographic follow-ups after retreatment were as followed: complete occlusion in four patients (cases 1, 2, 3, 4), obliteration of parent artery in one patient (case 5), showing no recurrence at 4–11 months (mean: 8.6 months). The mRS improved from 1 to 0 in four patients (cases 1, 2, 4, 5), from mRS 5 to 2 in one patient (case 6). Case 3 maintained mRS = 0 before and after retreatment. The last clinical follow-up, measured by mRS was excellent in five patients (cases 1–5), mild disability in only one patient (case 6). Retreatment results, angiographic and clinical follow-ups are shown in Table 3.

**Table 3:** Retreatment results and angiographic and clinical follow-up.

Patient NO.	Retreatment methods	Stent type, size (mm×mm) (number)	Immediate angiographic outcomes	Re-angiographic FU		Clinical FU	
				outcomes	time(mons)	time(mons)	mRS
**1**	SAC	EN 4.5×22 (1)	CO	CO	**10**	**51**	**0**
**2**	SAC	EN 4.5×22 (1)	NC	CO	**4**	**46**	**0**
**3**	coiling	—	CO	CO	**7**	**33**	**0**
**4**	SAC	EN 4.5×28 (1)	NC	CO	**11**	**19**	**0**
**5**	DS	EN 4.5×28 (2)	CMR	PAO	**11**	**18**	**0**
**6**	EOWAT	—	CO	**—**	**—**	**11**	**2**

DS = double stents; EOWAT = endovascular occlusion with aneurysm trapping; CMR = contrast medium retention; PAO = parent artery occlusion.

Representative cases are provided in figures 1–3. Other three cases are provided in figures S1, S2, S3.

## Discussion

As reconstructive endovascular modality, SAC of intracranial VADAs has been proven effective and feasible, especially when deconstructive treatment is contraindicated [7]–[9], [15]. Due to maintenance of patency of the parent artery, recurrence is a risk factor which influenced the prognosis of patients with intracranial VADAs after SAC [2], [11]. There have been some small case reports and case series about recurrence of intracranial VADAs after SAC [4], [14]–[16]. Post-treatment recurrence may be more dangerous in dissecting aneurysms than in berry aneurysms [4]. However, the retreatment and outcomes after recurrence have not been specifically studied. In our retrospective study, six recurrent intracranial VADAs after SAC were retreated with different endovascular modalities and followed up angiographically and clinically. From this case series, we tried to analyze the reasons and characteristics of recurrence, evaluate the strategies and outcomes of retreatment for recurrent VADAs after SAC.

### Reasons of recurrence

Recurrence has posed a great challenge to reconstructive endovascular treatment of intracranial VADAs [4], [14]–[16]. There is a 13–33% recurrence rate after endovascular treatment of VBDAs [4], [15], [16], [20]. On follow up angiography for 97 VBDAs in 89 patients after endovascular treatment, Kim et al. found that PICA origin involvement was the only independent risk factor for recurrence [4]. Persistent blood flow through the unprotected remnant dissecting aneurysm toward the PICA was believed to be the reason of recurrence. Differing from deconstructive treatment that obliterates the parent artery, SAC, as a reconstructive treatment modality, maintains the blood flow of parent artery increasing the possibility of recurrence.

Combined with related references and the experience gained from our case series we considered the recurrence probably due to the following reasons:

1. The stent did not cover the dissected length of the vessel completely [14]. Pathological research revealed that Internal Elastic Lamina (IEL) ruptures were longer than expected from angiographic results [18]. Among the total length of both intimal tears and medial defects, the mean length of proximal lesions from the center of the adventitial rupture was longer than that of distal lesions. With the increase of blood flow, from the defects of the intima, dissecting aneurysms can grow continuously. Although DSA still represents the gold standard in diagnostic imaging, Flat panel CTA such as Vaso CT, Dyna CT or Innova CT, MRI, MRA and HR-MRI have also demonstrated to be of value during initial treatment for a better understanding of the diseased segment of the parent vessel which should be covered with stents [27], [28].
2. Radial force of the Entreprise stent was insufficient. Self Expandable Stents (SES), have been broadly used in endovascular treatment of intracranial VADAs because of their flexibility [4], [9], [14]–[16]. Radial force against the vessel wall can promote the healing of the dissection. Meanwhile, SES can remodel the true lumen compressed by the dissecting aneurysm and the radial force may be less than the force of the blood flow within the pseudoaneurysms. Consequently, these types of stents may not attach or tack up the intimal flap to the parent vessel wall nor close the entrance of dissection.

Nowadays, no ideal stent seems to satisfy the demand of reconstructive endovascular treatment of intracranial VADAs. Because of the high porosity of the Entreprise stent, which is approximately 80% to 85% free metal to artery ratio, this self expandable stent can preserve the patency of perforating arteries, but the ability to divert the blood flow still poor in order to avoid recurrence [14]. These intracranial stents are not specifically designed to alter intra-aneurysmal hemodynamics. It is also difficult to navigate the tortuous vessels by using balloon expandable stents [21]. Newly developed flow diverters including Pipeline embolization device (PED) (Covidien/ev3 Irvine, CA, USA), SILK (BALT Extrusion, France) and the new Flow Re-direction Endoluminal Device FRED (MicroVention Inc. Tustin, CA, USA) devices, which have far less porosity than conventional neurovascular stents, may play a role in reconstructive treatment of intracranial dissecting aneurysms, by demonstrating favorable long-term clinical and angiographic outcomes with the ability to maintain parent artery and side-branch patency. There have been some severe complications previously reported such as rebleeding, side branch occlusion in posterior circulation dissecting aneurysms [13], [22], [23].To our knowledge, flow diverters have not been approved yet by the FDA for the treatment of vertebrobasilar dissecting aneurysms. There are few ongoing studies where the use of newest flow diverters is evaluated in the posterior circulation and there are also few recent case series published in the literature showing some potential good outcomes [24]. Flow diverters are not made widely available in our country for the moment.

3. Incomplete occlusion of intracranial aneurysms resulted in recurrence. Dense coil packing is crucial to decrease the rate of recurrence. But for dissecting aneurysm with irregular shape and complex geometry, plus fragile vessel wall, and without definite aneurysm neck, it is difficult and challenging to perform complete occlusion [20]. Persistent blood flow into dissecting aneurysm, not completely obliterated, will result in recurrence of the dissecting aneurysms.

In other words, in treating VADAs using SAC procedure if the flow diversion property of the stent and the packing density of the coils are not enough to reduce or exclude the blood flow from the dissecting aneurysm the recurrence of intracranial VADAs will occur.

### Strategies and outcomes of retreatment

With the development of intracranial stents, more and more intracranial VADAs are being treated with reconstructive endovascular modality based on increasing favorable results [8]–[13]. Meanwhile, the recurrence and retreatment should be evaluated. Retreatment modality of recurrent intracranial VADAs is determined by several factors including initial treatment modality, recurrence symptoms, recurrence types, neurovascular materials and the preference of the operators [16], [17], [19], [20]. In theory, reconstructive procedures are more reasonable than deconstructive ones because we cannot predict the destiny of collateral vessels for the rest of the patient's life. The prognosis is better with the preservation of the parent vessel [15]. However, for hemorrhagic recurrence, parent artery occlusion, which is deemed to be the most reliable option, can decrease the possibility of recurrence [4]. For recurrent intracranial VADAs which did not allowed for occlusion of the parent artery, reconstructive endovascular retreatment will be considered first [20]. In our case series, 5 (cases 1–5) of the 6 patients were retreated with reconstructive retreatment. Clinical follow-up was excellent in all five cases. The Enterprise stent used in our procedures is a self-expandable stent with high porosity. The flow diversion and radial force of a single stent were limited. Overlapping multiple stents technique has been shown to divert more blood flow from the dissecting aneurysm sac than a single stent by decreasing stent porosity, further straightening the parent artery, increasing stent thickness and subsequent neointimal endothelial formation [7], [25], [26]. In our study, retreatment with a second stent assisted coiling was performed in 3 patients (cases 1, 2, 4). The results suggest that retreatment with a second stent and better coiling, which improved radial force against vessel wall, decreased porosity, repaired the intimal flap, increased blood flow diversion, improved coil density and can gain a favorable result. Although angiographic follow up after 11 months revealed obliteration of the parent artery, in patient (case 5) there were no neurologic deficits. We analyzed that the mechanism was probably due to slow in-stent thrombosis and subsequent occlusion [16], [22].

Adequate flow diversion and dense packing are equally significant to avoid recurrence. But there are still several intracranial VADAs with unfavorable outcomes even after being treated by reconstructive endovascular techniques where adequate flow diversion and complete occlusion are seen because of the complexity and flow dynamics changes in dissecting aneurysms. In case 6, although three overlapping stents were deployed to try to remodel the blood flow and to achieve a complete occlusion in the initial treatment, recurrence still couldn't be avoided probably due to the severe deconstruction of the vessel wall. High resolution MR showed a huge VADA involving onion skin-like thrombus inside that aneurysm (Fig. 3 A). Because of severe recurrence symptom (sudden dyspnea) and regrowth of the original dissecting aneurysm we selected deconstructive endovascular retreatment modality for this patient. The improvement of clinical status on follow up showed that the retreatment modality was appropriate and effective for this kind of recurrent dissecting aneurysm.

In general, individualized strategies of retreatment should be done based on the initial treatment modalities and the characteristics of recurrence. We are optimistic about the retreatment of recurrent intracranial VADAs after SAC, based on our positive outcomes. But large case series and long-term follow up are necessary to draw a definite conclusion.

## Conclusions

Endovascular retreatment is feasible and effective for recurrent intracranial VADAs after SAC. Individualized strategies of retreatment should be enacted according to the reasons and characteristics of recurrence. But only proper design studies and large population data with proper follow-ups will help us to drive to that conclusion.

## Supporting Information

Figure S1 Case 2. A 49-year-old male was admitted with headache. DSA showed a left vertebral artery dissecting aneurysm distal to the origin of left PICA (1-1, 1-2). SAC were performed with complete occlusion (2-1, 2-2). Follow up angiography after seven months revealed regrowth of dissecting aneurysm from distal to the original dissecting aneurysm (3). Retreatment by stent assisted coiling (4) was performed with near complete occlusion (5-1, 5-2). Follow up angiography after 4 months of retreatment showed complete occlusion of recurrent dissecting aneurysm (6-1, 6-2). Follow up CTA after 22 months of retreatment showed no recurrence (7).
(TIF)

Figure S2 Case 4. A 49-year old male presented with headache and dizziness. MR imaging showed intramural hematoma and intimal flap (1-1, 1-2). Right vertebral angiograms showed a dissecting aneurysm involving PICA (2-1, 2-2, 2-3). SAC (3) were performed with partial occlusion (4-1, 4-2). Follow up angiography after six months revealed regrowth of the dissecting aneurysm (5-1, 5-2, 5-3). Retreatment by stent assisted coiling (6) was performed with near complete occlusion (7). Follow up angiography after 11 months of retreatment showed complete occlusion of recurrent dissecting aneurysm (8-1, 8-2).
(TIF)

Figure S3 Case 5. A 53-year old male presented with headache and dizziness. MR imaging showed intramural hematoma and intimal flap (1-1, 1-2). Left vertebral angiograms showed a dissecting aneurysm (2-1, 2-2). SAC (3) were performed with partial occlusion (4). Follow up angiography after five months revealed recanalization of the dissecting aneurysm (5-1, 5-2, 5-3). Retreatment by double stent (6) was performed with contrast medium retention (7-1, 7-2). Angiographic follow up after 11 months revealed obliteration of the parent artery (8-1, 8-2, 8-3).
(TIF)
