# Supplementary figures and images for: Retreatment and Outcomes of Recurrent Intracranial Vertebral Artery Dissecting Aneurysms after Stent Assisted Coiling: A Single Center Experience
Source: PLoS One. 2014 Nov 13;9(11):e113027. doi: 10.1371/journal.pone.0113027 (PMC4231114; doi:10.1371/journal.pone.0113027)

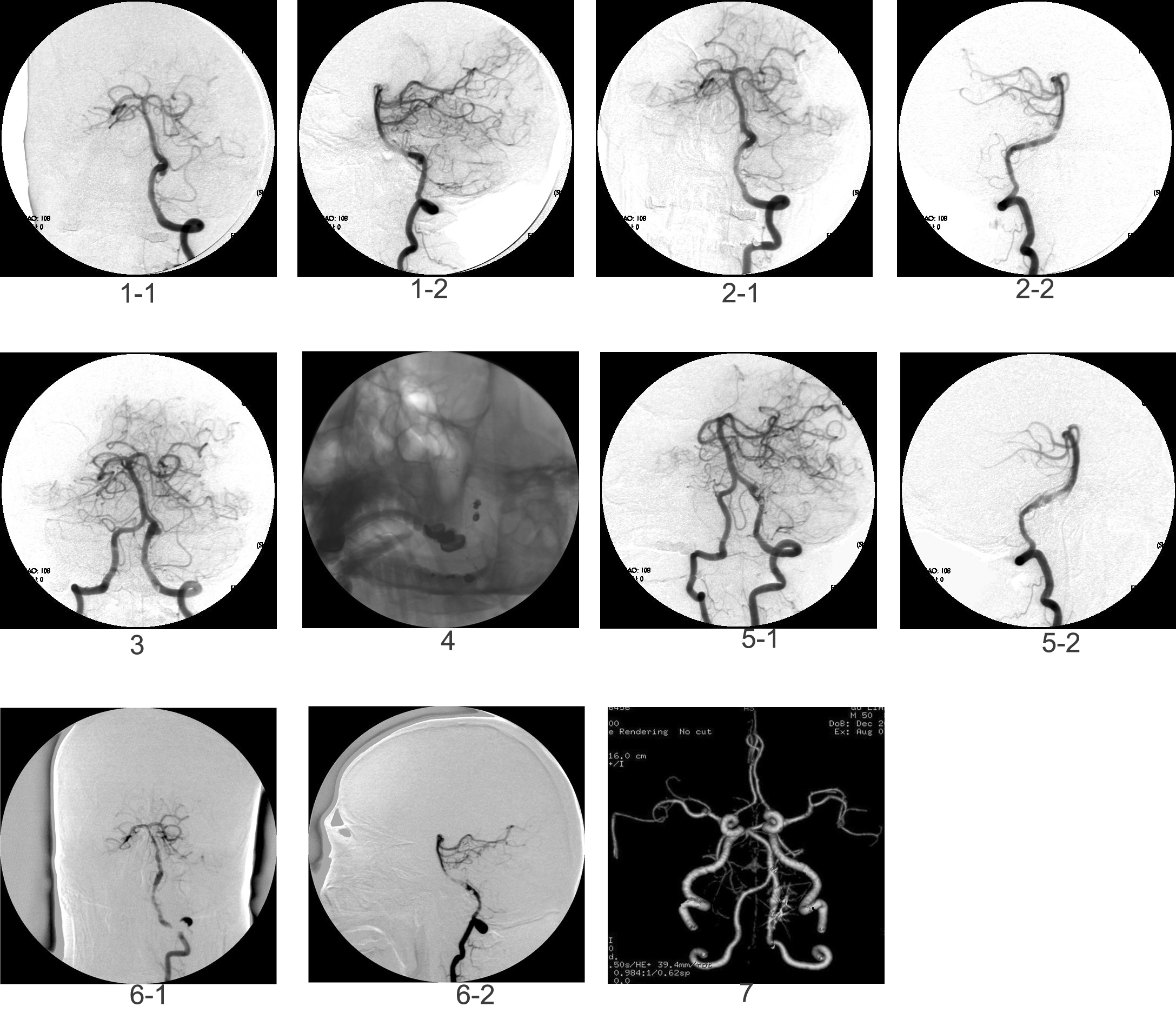

Supplement: Figure S1 — Case 2. A 49-year-old male was admitted with headache. DSA showed a left vertebral artery dissecting aneurysm distal to the origin of left PICA (1-1, 1-2). SAC were performed with complete occlusion (2-1, 2-2). Follow up angiography after seven months revealed regrowth of dissecting aneurysm from distal to the original dissecting aneurysm (3). Retreatment by stent assisted coiling (4) was performed with near complete occlusion (5-1, 5-2). Follow up angiography after 4 months of retreatment showed complete occlusion of recurrent dissecting aneurysm (6-1, 6-2). Follow up CTA after 22 months of retreatment showed no recurrence (7). (TIF) [file pone.0113027.s001.tif]

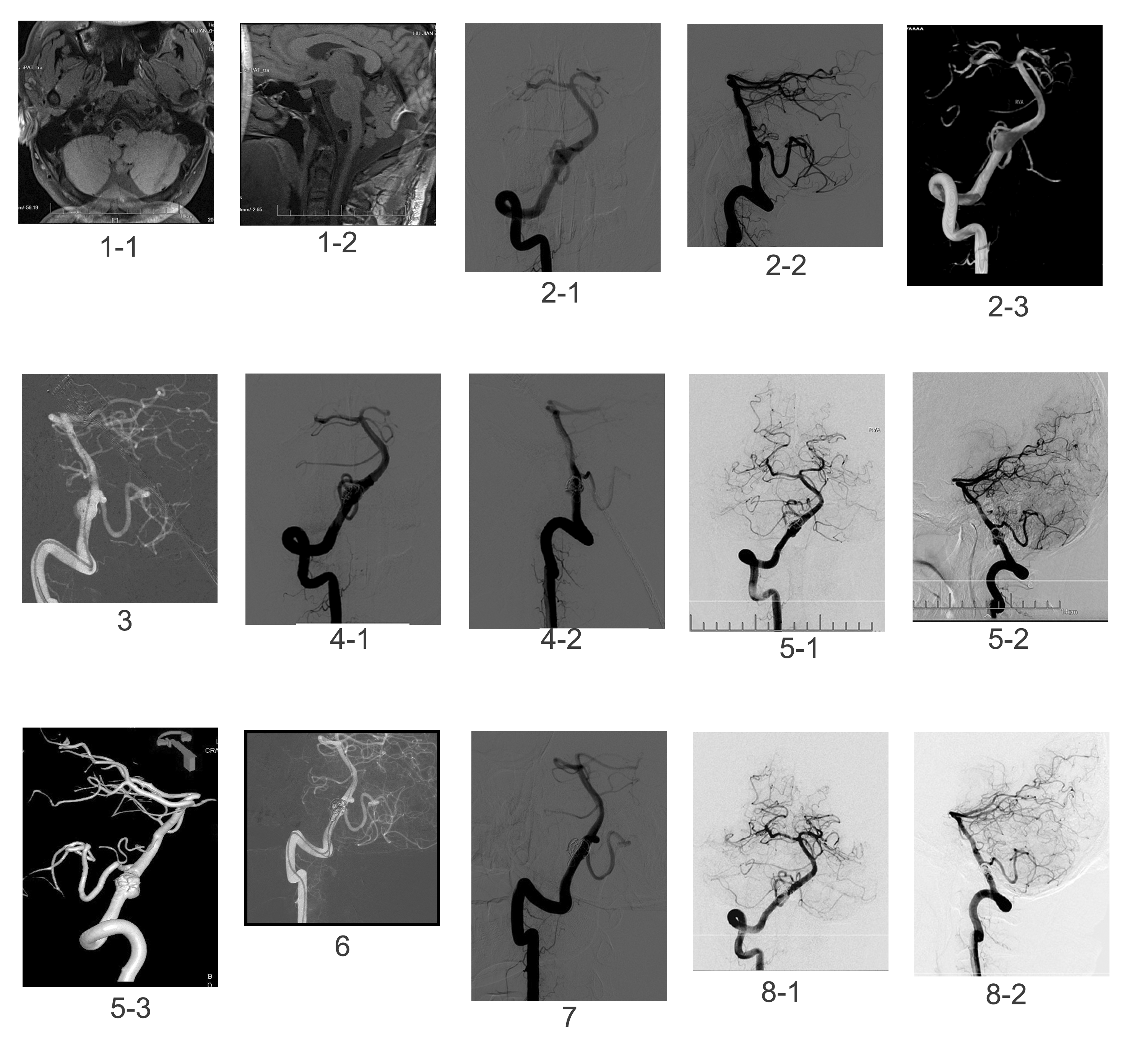

Supplement: Figure S2 — Case 4. A 49-year old male presented with headache and dizziness. MR imaging showed intramural hematoma and intimal flap (1-1, 1-2). Right vertebral angiograms showed a dissecting aneurysm involving PICA (2-1, 2-2, 2-3). SAC (3) were performed with partial occlusion (4-1, 4-2). Follow up angiography after six months revealed regrowth of the dissecting aneurysm (5-1, 5-2, 5-3). Retreatment by stent assisted coiling (6) was performed with near complete occlusion (7). Follow up angiography after 11 months of retreatment showed complete occlusion of recurrent dissecting aneurysm (8-1, 8-2). (TIF) [file pone.0113027.s002.tif]

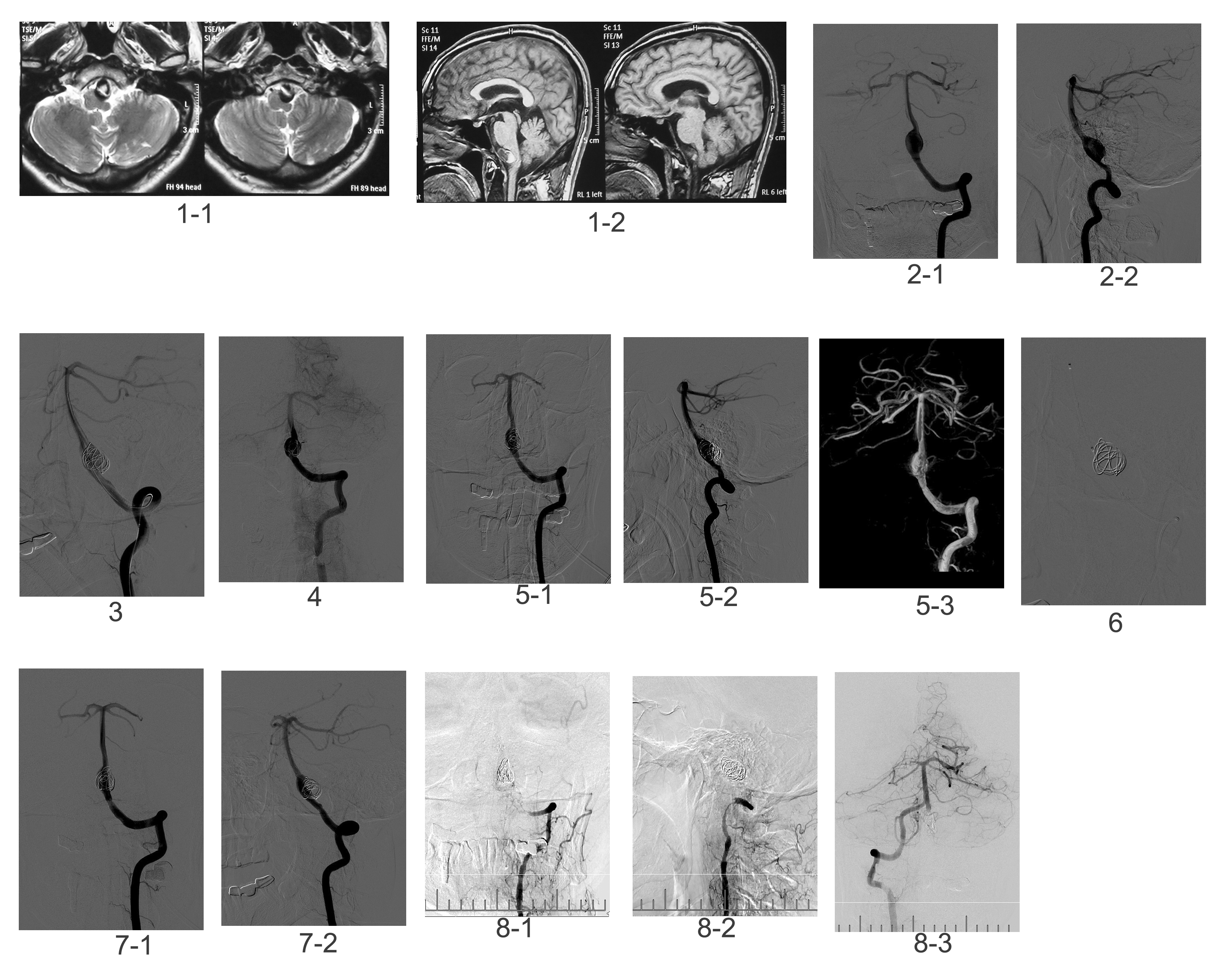

Supplement: Figure S3 — Case 5. A 53-year old male presented with headache and dizziness. MR imaging showed intramural hematoma and intimal flap (1-1, 1-2). Left vertebral angiograms showed a dissecting aneurysm (2-1, 2-2). SAC (3) were performed with partial occlusion (4). Follow up angiography after five months revealed recanalization of the dissecting aneurysm (5-1, 5-2, 5-3). Retreatment by double stent (6) was performed with contrast medium retention (7-1, 7-2). Angiographic follow up after 11 months revealed obliteration of the parent artery (8-1, 8-2, 8-3). (TIF) [file pone.0113027.s003.tif]
